# Metabolic heterogeneity signature of primary treatment-naïve prostate cancer

**DOI:** 10.18632/oncotarget.15237

**Published:** 2017-02-09

**Authors:** Dong Lin, Susan L. Ettinger, Sifeng Qu, Hui Xue, Noushin Nabavi, Stephen Yiu Chuen Choi, Robert H. Bell, Fan Mo, Anne M. Haegert, Peter W. Gout, Neil Fleshner, Martin E. Gleave, Michael Pollak, Colin C. Collins, Yuzhuo Wang

**Affiliations:** ^1^ The Vancouver Prostate Centre, Vancouver General Hospital and Department of Urologic Sciences, The University of British Columbia, Vancouver, British Columbia, Canada; ^2^ Department of Experimental Therapeutics, BC Cancer Research Centre, Vancouver, British Columbia, Canada; ^3^ Division of Urology, University of Toronto, Department of Urology, University Health Network, Princess Margaret Hospital, Toronto, Ontario, Canada; ^4^ Lady Davis Research Institute and McGill University, Montreal, Quebec, Canada

**Keywords:** prostate cancer, tumour heterogeneity, metabolic heterogeneity, patient-derived xenografts

## Abstract

To avoid over- or under-treatment of primary prostate tumours, there is a critical need for molecular signatures to discriminate indolent from aggressive, lethal disease. Reprogrammed energy metabolism is an important hallmark of cancer, and abnormal metabolic characteristics of cancers have been implicated as potential diagnostic/prognostic signatures. While genomic and transcriptomic heterogeneity of prostate cancer is well documented and associated with tumour progression, less is known about metabolic heterogeneity of the disease. Using a panel of high fidelity patient-derived xenograft (PDX) models derived from hormone-naïve prostate cancer, we demonstrated heterogeneity of expression of genes involved in cellular energetics and macromolecular biosynthesis. Such heterogeneity was also observed in clinical, treatment-naïve prostate cancers by analyzing the transcriptome sequencing data. Importantly, a metabolic gene signature of increased one-carbon metabolism or decreased proline degradation was identified to be associated with significantly decreased biochemical disease-free patient survival. These results suggest that metabolic heterogeneity of hormone-naïve prostate cancer is of biological and clinical importance and motivate further studies to determine the heterogeneity in metabolic flux in the disease that may lead to identification of new signatures for tumour/patient stratification and the development of new strategies and targets for therapy of prostate cancer.

## INTRODUCTION

Prostate cancer is a clinically heterogeneous disease covering a wide variety of phenotypes, ranging from indolent disease to aggressive lethal disease. At present, there is a lack of methods for reliably identifying such phenotypes, rendering use of first-line androgen ablation therapy a challenge with a risk of over- or under-treatment [1]. As such, there is an urgent need for signatures to stratify indolent from aggressive prostate cancer which could be developed through identification of differences in energy metabolisms of prostate cancer subtypes [2].

Reprogramming energy metabolism is an important hallmark of cancer [3, 4] and abnormal metabolic characteristics of cancers have been implicated as potential diagnostic/prognostic biomarkers [5–9]. Thus in contrast to normal resting cells whose energy production is mainly based on mitochondrial oxidative phosphorylation, the majority of cancer cells rely on aerobic glycolysis coupled to increased glucose uptake and lactic acid production/secretion (the Warburg effect) [3, 7]. The augmented glucose consumption can be observed using (^18^F)-fluoro-2-deoxyglucose positron emission tomography (FDG-PET) [10, 11]. However, prostate cancers are characterized by a lack of increased glycolysis rendering FDG-PET less effective in imaging this malignancy [12], (although a small group of tumours do utilize aerobic glycolysis [9]). Instead, prostate cancers have abnormal metabolisms for citrate and choline [13–15] and are characterized by increased levels of choline-containing compounds (ChoCCs). These compounds have been linked to increased cell proliferation and survival [16]. Tracing metabolites with (^11^C)- and (^18^F)-labeled choline derivative-based PET and PET/CT scans is increasingly used in the clinic. Together, these glucose and choline-based imaging studies demonstrated the metabolic heterogeneity of clinical prostate cancer [17, 18]. However, it is not clear whether prostate cancer subtypes are metabolically different, or whether metabolic heterogeneity (intra-tumoural and inter-tumoural) can affect tumour response to treatment. In view of this, a better understanding of metabolic heterogeneity of prostate cancer at a molecular level will provide mechanistic insights into disease progression and may lead to identification of novel diagnostic/prognostic signatures for stratification of patients’ prostate cancers.

To focus on the metabolic heterogeneity of prostate cancer cells and avoid complication from the stromal compartment, we used our unique panel of transplantable, patient-derived xenograft (PDX) models, derived from hormone-naïve prostate cancer tissues (www.livingtumorlab.com) [19, 20]. Using these hormone-naïve prostate cancer PDX models and clinical samples, we, for the first time, demonstrated the heterogeneity of expression of genes involved in cellular energetics and macromolecule biosynthesis and identified an inter-tumour metabolic heterogeneity signature in clinical samples that is associated with patients’ prognosis.

## RESULTS

### A panel of PDX models of hormone-naïve prostate cancers demonstrates heterogeneity of expression of genes involved in energy metabolism and recapitulates metabolic heterogeneity of clinical samples

A panel of 11 well-characterized LTL (Living Tumour Laboratory) prostate cancer PDX-models that are derived from hormone-naïve prostate cancer tissues, presents a unique opportunity for focusing on metabolic alterations in prostate cancer cells by analyzing human genes (while stromal genes are of mouse origin) *in vivo* [19]. To investigate whether these hormone-naïve PDX models present with molecular metabolic heterogeneity, transcriptome microarray data were generated and cluster analysis was performed using mean pathway scores (derived from per gene z-scores normalized to cancer mean and standard deviation, Supplementary Table 2). As shown in Figure 2, the metabolic pathway scores demonstrated variation among these models. For example, four tumours had increased pathway scores for glycolysis, while seven other tumours had decreased scores for this pathway. Heterogeneity was evident for all pathways.

**Figure 1:**
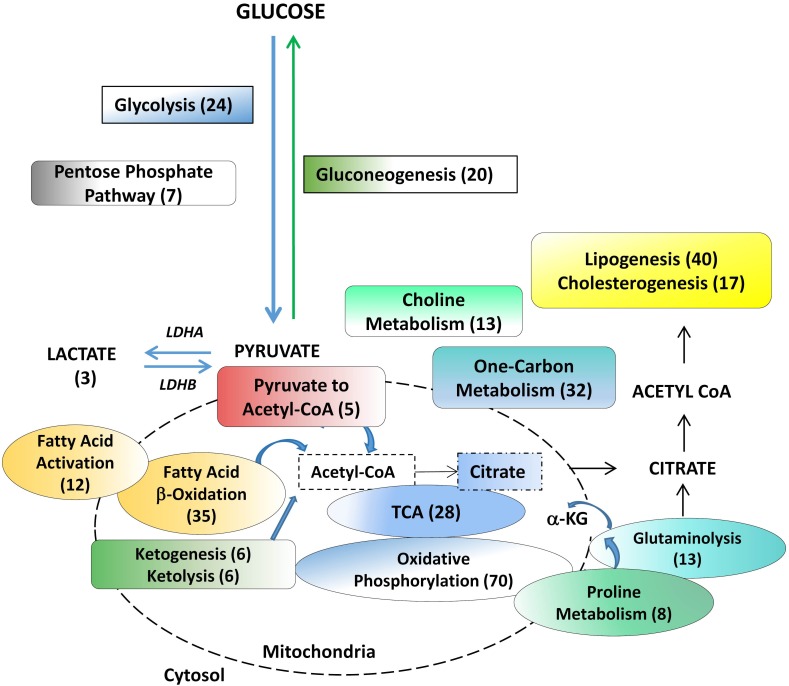
Metabolic pathways assessed in benign tissue, primary hormone-naive prostate cancer and patient-derived hormone-naïve xenograft models Products of choline metabolism are linked to cell proliferation and are used for prostate cancer diagnosis. The remaining pathways are involved in biosynthesis of macromolecules and bioenergetics. Numbers in brackets indicate the number of genes analyzed within a particular pathway. LDHA (Lactate Dehydrogenase A), a-KG (alpha ketoglutarate).

**Figure 2:**
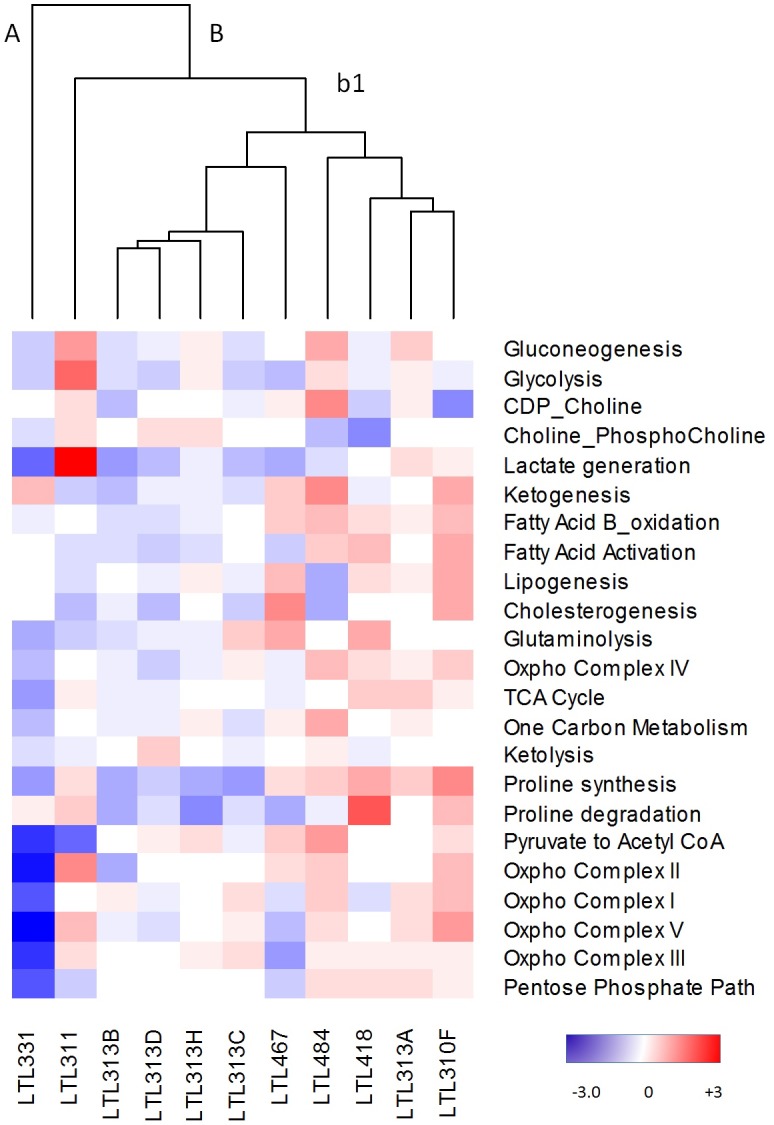
Metabolic heterogeneity in the PDX model Cluster analysis of metabolic pathway scores from the PDX model (LTL). Z-scores were normalized to all tumours (mean/standard deviation) for each gene within a pathway. Average z-scores were then used to generate pathway scores. Red, upregulated; blue, downregulated; white, no change from tumour average. A, B, and b1 represent various arms of the dendogram.

**Figure 3:**
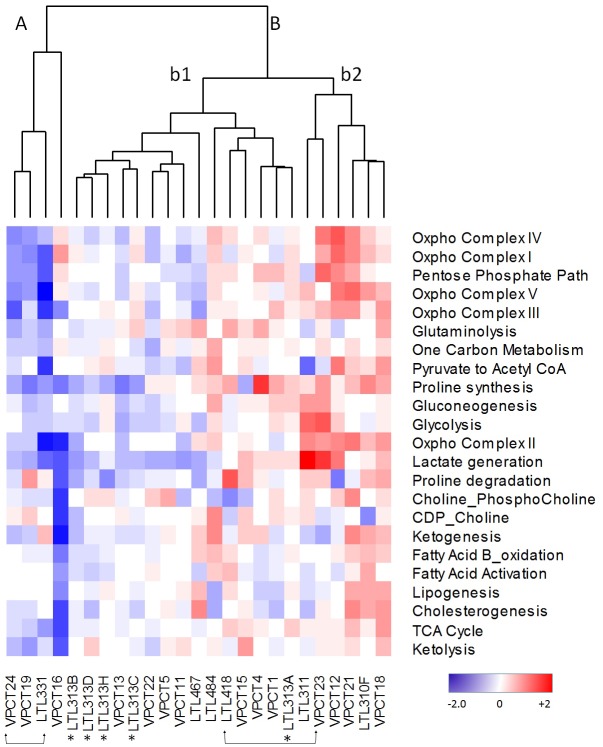
Cluster analysis of metabolic pathway z-scores derived from DESEq RNA sequencing expression data from the VPC Cohort (VPC) compared to z-scores derived from quantile normalized microarray expression data from the PDX cohort (LTL) Red, upregulated; blue, downregulated; white, no change from tumour average. Two patient/LTL PDX pairs are indicated by arrow brackets. *Five LTL PDXs derived from needle biopsy specimens of 5 different foci of a patient's primary prostate cancer tissue.

To investigate whether such heterogeneity of metabolic gene expression observed in the PDX models represents the clinical situation, we analyzed transcriptome sequencing data of treatment-naïve, primary prostate cancer specimens of the VPC cohort (*n* = 14). The VPC prostate cancer per-gene z-scores were normalized to cancer mean and standard deviation, to generate mean pathway scores. The cluster analysis combining PDX models and VPC clinical samples showed that (in Figure 3), all the PDX models were clustered into different arms as observed for the clinical samples, suggesting that this set of PDX models recapitulates the heterogeneous metabolic nature of the clinical samples. In addition, the matched PDX/parental tissue pairs LTL-331/VPCT24 and LTL418/VPCT23, clustered closely together, as seen in dendrogram arms A and B respectively, illustrating the fidelity of the PDX models (arrow brackets). Interestingly, the LTL-313 xenograft lines, which had been derived from needle biopsies at five different foci of one patient's primary prostate cancer (asterisk), show a diverse heterogeneity signature in each focus. The observed differences between the five cores from which these models originate suggest the existence of diverging events that are continually associated with the development of intra-tumoural metabolic heterogeneity of prostate cancer. In summary, we documented molecular evidence of cancer metabolic heterogeneity in PDX models and clinical hormone-naïve prostate cancer samples. Importantly, the metabolic heterogeneity discovered in PDX models was validated in clinical prostate cancer samples.

### Change in expression of genes involved in metabolism is consistent with the metabolomic changes in prostate cancer

To determine whether the gene expression involved in metabolic pathways is consistent with the metabolic properties of prostate cancer cells, we first analyzed the transcriptomic differences, between benign and treatment-naïve prostate tumours, of genes involved in metabolic pathways and we then explored the correlation with clinical metabolomics. A comparison of the MSKCC dataset [21] consisting of 28 benign prostate tissues and 112 prostate adenocarcinomas from treatment-naïve patients, showed significantly increased expression of genes involved in choline/phosphocholine generation (*p* < 0.0002) and in proline synthesis (*p* < 0.0029) (Table 2) in tumours compared to benign tissue. These results reflect the findings of several recent clinical metabolomics studies reporting increased choline metabolites and increased proline [22–25] in prostate cancer compared to benign tissue.

**Table 1:** VPC prostate cancer cohort clinical details

VPC ID	Tumour or Benign	Age at Dx	PSA at Dx	Gleason score	PSA recurrence months to	Follow up (months post-surgery)	ETS status
VPCT1	T	74	2.1	9 (4+5) N		123	ERG
VPCT4	T	65	17.0	9 (4+5)	Y (nrn)	71	negative
VPCB4	B						n/a
VPCT5	T	71	8.0	8 (4+4)	Y (34)	94	ERG
VPCT11	T	65	8.1	7 (3+4)	N	83	ERG
VPCB11	B						n/a
VPCT12	T	64	1.37	9 (4+5)	Y (30)	66	ETV1
VPCT13	T	57	31.0	9 (4+5)	Y (28)	48	ERG
VPCB13	B						n/a
VPCT15	T	69	15.07	7 (4+3)	Y (nrn)	41	negative
VPCT16	T	45	6.3	10 (5+5)	Y (3)	45	negative
VPCT18	T	59	10.1	8 (4+4)	Y (15)	46	ETV1
VPCT19	T	53	7.7	8 (4+4)	N	18 (LTFU)	ERG
VPCT21	T	70	65.3	9 (4+5)	Y (nrn)	5 (LTFU)	ERG
VPCT22	T	47	4.2	9 (5+3)	Y (55)	59	ERG
VPCT23	T	63	22	10 (5+5)	Y (nrn)	40	ETV1
VPCT24	T	64	17	9 (5+4)	Y (nrn)	49	ERG

The prostate cancer cohort of the Vancouver Prostate Centre (VPC) consisted of 14 untreated primary tumours and 3 matched benign prostate tissues [44]. Abbreviations: T, tumour; B, Benign; Dx, diagnosis; PSA, prostate specific antigen; nrn, never reached nadir; N, no; Y, yes; LTFU, lost to follow-up. There were no differences in stromal content across the cohort based on comparisons of desmin and vimentin expression (fibroblasts) [44].

**Table 2:** MSKCC cohort: comparison of metabolic pathway scores between hormone-naïve prostate cancer and benign tissue

Metabolic Pathway	t-test
Choline_PhosphoCholine	0.0002 (upregulated)
Proline Synthesis	0.0029 (upregulated)
Pyruvate to Acetyl CoA	0.0092 (downregulated)
Lipogenesis	0.0580 (downregulated)
Proline Degradation	0.0708 (downreguated)
Cholesterogenesis	0.0983
Fatty Acid B-oxidation	0.1059
TCA Cycle	0.1086
Oxpho II Complex	0.1096
CDP-Choline	0.1365
Oxpho IV Complex	0.1618
Fatty Acid Activation	0.2099
Glycolysis	0.2573
One-carbon metabolism	0.3883
Oxpho I Complex	0.4015
Lactate generation	0.4800
Gluconeogenesis	0.5183
Glutaminolysis	0.5615
Ketolysis	0.5985
Ketogenesis	0.6802
Oxpho III Complex	0.7039
Oxpho V Complex	0.7095
Pentose Phosphate Pathway	0.7922

Using the Students t-test (*p*<0.05) prostate cancer (*n*=112) log2 gene expression for each pathway was compared to benign tissue (*n*=28) log2 gene expression for each pathway.

Interestingly, expression of genes involved in pyruvate to acetyl-CoA formation was significantly decreased (*p* < 0.0092). As expected, there were no significant differences in the expression of glycolysis pathway genes (i.e. upstream of the PDH complex) between benign tissue and cancer (Table 2), which is consistent with previous reports and reflects the clinical finding that FDG-PET is less effective in imaging prostate malignancy [15] than other cancers.

### Gene expression of a metabolic signature demonstrates heterogeneity in clinical samples and clinical outcome of prostate cancer patients

**Figure 4:**
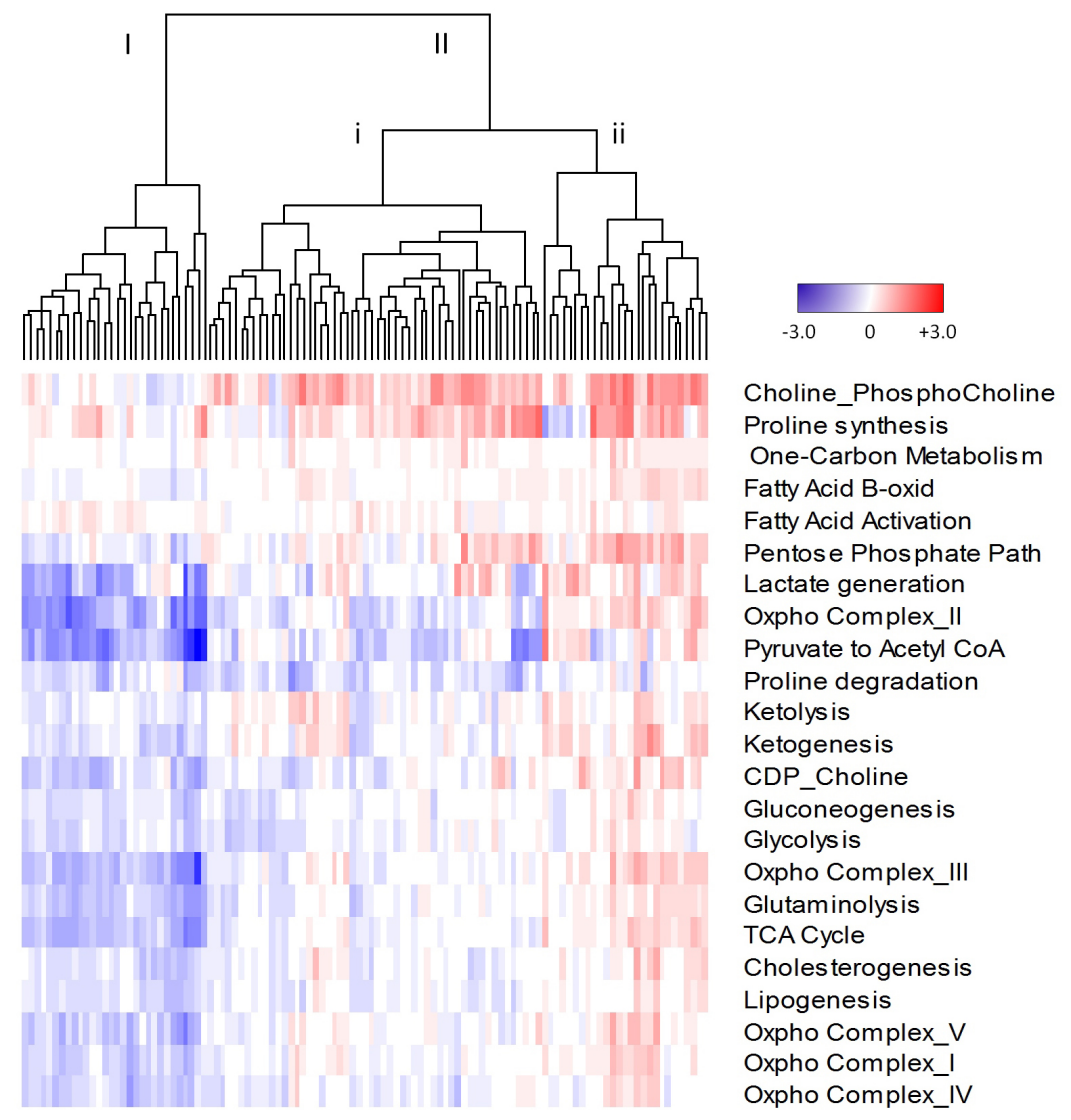
Cluster analysis of metabolic pathway scores derived from quantile normalized microarray expression data from the MSKCC cohort Z-scores were normalized to benign mean and standard deviation for each gene within a pathway. Average z-scores were then used to generate pathway scores. Dendrogram arms labelled I, II, I and ii, represent various groups of tumours. Red, upregulated; blue, downregulated; white, no change from benign.

To further validate our finding of metabolic heterogeneity of treatment-naïve prostate tumours and explore its clinical significance, we analyzed the correlation of our heterogeneous metabolic signature with clinical outcome in the MSKCC dataset [21]. As shown in Figure 4, heterogeneity of metabolic pathways between tumours is evident. Dendrogram arms I and II indicate two broadly different groups of tumours. Group II shows further heterogeneity as indicated by dendrogram arms (i and ii). The MSKCC validation cohort showed significant heterogeneity (Wilcoxon Rank test, *p* < 0.05) of metabolic pathways involved in cholesterogenesis, choline metabolism, gluconeogenesis, glutaminolysis, glycolysis, ketogenesis, lipogenesis, oxidative phosphorylation involving complexes I, IV and V, the TCA cycle and one-carbon metabolism (Supplementary Table 4). Pathways with less heterogeneity also occurred, including fatty acid activation and ketolysis. Interestingly, proline synthesis and proline degradation pathways showed opposite directions of expression. The proline synthesis pathway was consistently upregulated and proline degradation pathway was downregulated compared to benign tissue controls, which together supports metabolomics results [17] of increased proline content in aggressive prostate cancer. This is also consistent with results in the previous section (Table 2) where we showed that the proline synthesis pathway score was significantly increased in cancer compared to benign tissue (*p* < 0.0029).

**Figure 5:**
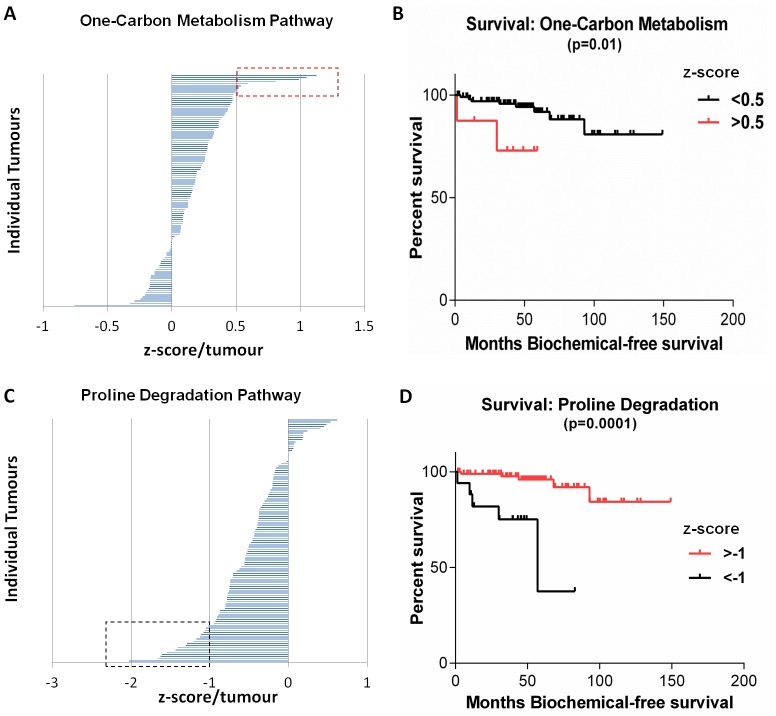
Metabolic pathways within the MSKCC Cohort associated with significantly decreased survival **(A**) Tumours over-expressing genes in the one-carbon metabolism pathway were associated with significantly decreased biochemical-free survival (**B**), *p* = 0.01. **(C**) Tumours with decreased expression of genes regulating proline degradation were associated with significantly decreased biochemical-free survival (**D**), *p* = 0.0001. Red and black boxes indicate tumour outliers ( > 2 Standard deviations).

To determine the clinical relevance of the observed metabolic heterogeneity we carried out survival curve analyses (5 year-overall survival as well as biochemical recurrence-free survival) for the MSKCC cohort (Supplementary Table 5). Patients with elevated expression levels of genes involved in one-carbon metabolism, compared to controls ( > 2 Standard Deviations, Supplementary Table 6), showed significantly poorer biochemical-free survival compared to patients with lower expression of these genes (*p* < 0.01) (Figure 5A-5B). Additionally, patients with low expression levels of genes involved in proline degradation, compared to controls (Supplementary Table 6), had significantly poorer biochemical-free survival compared to patients with higher expression levels (Figure 5C-5D). Therefore, increased one-carbon metabolism or decreased proline degradation in these patients was associated with significantly decreased survival, suggesting these metabolic changes form clinically important metabolic signatures. There was a likely-correlation (Spearman) of one carbon metabolism and proline degradation pathway scores with Pathology stage (*p* = 0.06 and *p* = 0.08 respectively). This suggests that increased one carbon metabolism or decreased proline degradation (therefore increased proline levels) is observed in more aggressive tumours compared to less aggressive tumours. Such increased one carbon metabolism or decreased proline degradation signature was also observed in 50% or 75% (respectively) of PDX models with metastatic ability suggesting a predictive value of aggressiveness.

## DISCUSSION

Whereas the genomic and transcriptomic heterogeneity of prostate cancer is well documented [26], less is known about metabolic heterogeneity of this malignancy and its relevance to malignant progression. Studies of the heterogeneity of prostate cancer, in particular hormone-naïve cancers, have been impeded by a lack of clinically relevant experimental models. Commonly used prostate cancer xenograft models based on cultured human prostate cancer cells do not accurately recapitulate the tumour heterogeneity, nor the cancer-stroma architecture of the original cancer specimens from which the cell lines were derived [19, 20]. Importantly, the majority of prostate cancer cell lines and xenograft models used were derived from advanced metastatic castration-resistant cancer, rather than untreated hormone-naïve cancer. In this study, we demonstrated the heterogeneous expression of cancer cell genes involved in energy metabolisms in 11 patient-derived prostate cancer PDX models derived from hormone-naïve tumours. These models belong to a previously produced panel of prostate cancer PDX models [19, 20] and to our knowledge currently form the largest collection of hormone-naïve prostate cancer PDX models in the field. Furthermore, the metabolic heterogeneity signature in these models suggests, for the first time, that a panel of PDX models can recapitulate heterogeneous metabolic signatures observed in clinical samples. The metabolic program that correlated with poor prognosis in the MSKCC dataset was associated with metastasis in PDX models. As such, these models provide a valuable platform for studying metabolic heterogeneity.

**Figure 6:**
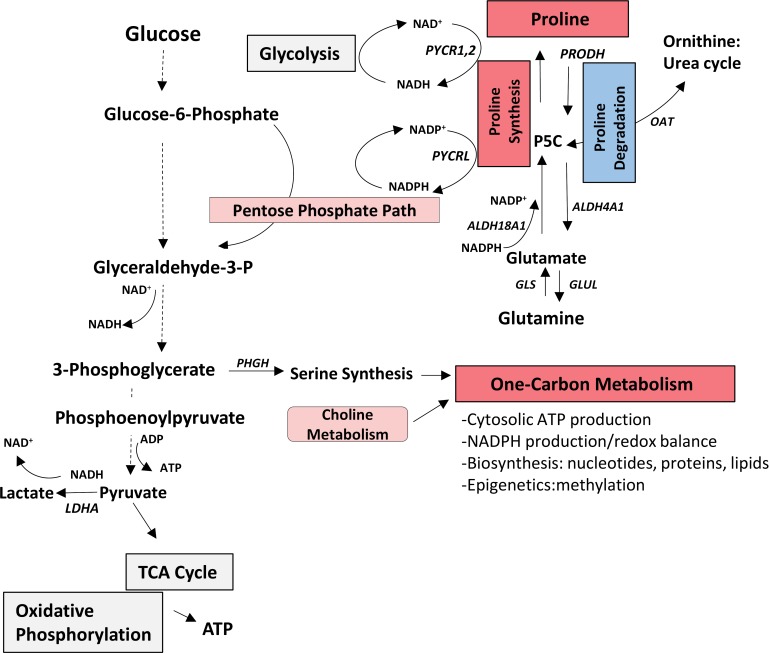
Summary of heterogeneity of expression of genes related to core metabolic pathways involved in cellular energetics and macromolecule biosynthesis in hormone-naive prostate cancer The one-carbon metabolism pathway score was significantly increased (red) and the proline degradation pathway score was decreased (blue) compared to benign and both were associated with decreased biochemical-free survival in the MSKCC subset. It has been reported that the one-carbon metabolism and proline metabolism pathways interconnect with glycolysis and pentose phosphate pathway to provide ATP, and NADH and NADPH (mediators of electron transfer for energy, reductive biosynthesis and redox defense) [8, 9, 42]. (Pink indicates upregulated pathways in MSKCC subset; grey indicates other heterogeneous pathways). ALDH18A1, Pyrroline-5-carboxylase synthase; ALDH4A1, Pyrroline-5-carboxylase dehydrogenase; GLS, Glutaminase; GLUL, Glutamine synthase; P5C, Pyrroline-5-carboxylase; LDHA, Lactate Dehydrogenase A; OAT, Ornithine aminotransferase; PHGH, Phosphoglycerate Dehydrogenase; PRODH, Proline Dehydrogenase; PYCR1,2,L, Pyrroline-5-carboxylate reductase 1,2,L.

Using RNA sequencing strategies and microarrays to investigate heterogeneity of expression of genes involved in cellular energetics and macromolecular biosynthesis pathways, the present study documents inter-tumour metabolic expression heterogeneity of several pathways/sub-pathways in PDX models and in two clinical cohorts of, treatment-naïve prostate cancers as summarized in Figure 6. Consistent with these findings, heterogeneity of several metabolic pathways has been observed in a meta-analysis of datasets of 22 different tumour types [27]. Importantly, we identified metabolic gene signatures of increased one-carbon metabolism or decreased proline degradation associated with significantly decreased biochemical disease-free survival of patients. One limitation of this study is the lack of metabolomics validation. Further integrated analysis of transcriptomic and metabolomics data and functional studies will provide more insights regarding the significance of metabolic heterogeneity in prostate cancer.

It is widely reported that cancer cells can direct glucose metabolism toward aerobic glycolysis [28]. However, consistent with our own results, emerging evidence suggests that prostate cancer cells can also direct glucose towards another ATP-generating pathway involving 3-phosphoglycerate conversion to serine through phosphoglycerate dehydrogenase (PHGDH), resulting in activation of one-carbon metabolism [29–31]. One-carbon metabolism facilitates NADPH generation [32] and the anabolic synthesis of amino acids, proteins, nucleotides and phospholipids, and has a role in the maintenance of redox balance and methylation reactions involved in post-translational modifications [33, 34] and so may be a potential driver of oncogenesis. Indeed, methotrexate, an inhibitor of the one-carbon cycle, is used as an anti-cancer agent and preclinical studies are evaluating several other enzymes in this pathway [33]. Importantly, however, methotrexate has recently been associated with accelerated progression of a previously indolent prostate cancer [35]. An alternative therapeutic, metformin, has also been shown to indirectly inhibit one-carbon metabolism [36] and is being tested in preclinical trials for prostate cancer [37–39]. Several agents targeting metabolic enzymes exist for other metabolic syndromes that could be re-purposed for prostate cancer [37].

Proline metabolism promotes cancer cell proliferation and energy production [40]. The cycling of proline catabolism and proline synthesis acts as a redox shuttle between the mitochondria and cytosol to maintain redox homeostasis [41, 42]. Furthermore, increased proline biosynthesis contributes to cancer cell growth by providing NAD+/NADP+ molecules to the pentose phosphate pathway and to glycolysis [40]. Here we show that a decreased pathway score for proline degradation is associated with significantly decreased biochemical disease-free survival of prostate cancer patients. Concomitantly, the expression of the proline synthesis pathway genes was consistently increased in prostate tumours implying a strong propensity of these tumours to activate the proline synthesis cycle. In support of our findings, LC/GC-MS metabolomic profiling identified increased proline levels with increased progression of prostate cancer [22–25].

Increased proline synthesis has also been reported as the major metabolic shift in metastatic breast cancer cells, in melanoma cell lines compared to melanocytes and in ovarian cancer cells (OVCAR3) compared to ovarian cancer stem cells [40]. Furthermore, a recent study of 133 metabolic genes identified five genes whose siRNA-induced knockdown resulted in strong growth-inhibitory effects on breast cancer cells. Included were PYCR1 involved in proline synthesis and the serine biosynthesis gene, PDGDH, involved in one-carbon metabolism [43].

In conclusion, we have, for the first time, demonstrated heterogeneity of expression of genes related to metabolic pathways in both hormone-naïve prostate cancer PDX models and clinical samples and provided insight into the metabolic heterogeneity of hormone-naïve prostate cancer associated with patient clinical outcome. These results not only suggest the potential of a metabolic heterogeneity signature as a biomarker for patient risk stratification, but also motivate further work to determine whether the observed differences in expression of genes involved in metabolic pathways translate into differences in metabolic flux. A better understanding of metabolic heterogeneity of hormone-naïve prostate cancer will lead to the identification of new signatures for patient stratification and the development of new strategies and targets for therapy of prostate cancer.

## MATERIALS AND METHODS

### Patient-derived xenograft (PDX) models - discovery cohort

Transplantable xenograft lines of patients’ prostate cancer tissues were developed by grafting of fresh cancer tissue (obtained from needle biopsies), and serial transplantation, into the subrenal capsule (SRC) graft site of male NOD/SCID mice supplemented with testosterone as previously described [19]. Animal care and experiments were carried out in accordance with the guidelines of the Canadian Council on Animal Care.

### Gene expression analysis of xenografts using RNA microarray analysis

RNA microarray analysis of PDX tissue was performed as previously described [19]. Data are available at GEO accession number GSE41193. Total RNA samples were prepared and processed using Agilent's One-Color Microarray-Based Gene Expression Analysis Low Input Quick Amp Labelling v6.0. An input of 100 ng of total RNA was used to generate cyanine-3-labeled cRNA. Samples were hybridized on Agilent SurePrint G3 Human GE 8×60K Microarray (Design ID, 028004). Arrays were scanned with the Agilent DNA Microarray Scanner at 3-μm scan resolution and data were processed with Agilent Feature Extraction 11.0.1.1. The processed signal was quantile normalized with Agilent GeneSpring 12.0.

### Validation cohorts

The prostate cancer cohort of the Vancouver Prostate Centre (VPC) consisted of 14 untreated primary tumours and 3 matched benign prostate tissues [44] as described in Table 1. Gleason scores ranged from 7-10 and samples consisted of ≥ 70% of malignant glands. The MSKCC cohort consisted of 112 untreated primary prostate tumours and 28 benign prostate tissues [21]. Data are available at GEO accession number GSE21032.

### Gene expression analysis of tumor sections using RNA sequencing

VPC clinical tumour sections were processed and RNA sequencing was performed at the BCCA Michael Smith Genome Sciences Centre according to standard protocol as previously described [44]. Briefly, RNA-seq data reads were first mapped onto the hg19 human reference genome and exon-exon junctions by splice-aware aligner STAR [45], using known gene model annotation from Ensembl Release 75. Reads with an unmapped mate or multi-mapped location were filtered out using Bam Tools [46]. Data are available at GEO accession number GSE55016.

Using aligned RNA-seq reads, gene expression profiles for each sample were calculated on the basis of gene annotation from Ensembl Release 75. Only reads that were unique to one gene and exactly corresponded with the structure of the gene were counted for the corresponding genes by using tool HTSeq [47]. In order to eliminate the variance of sequencing depth among samples, the raw read counts were normalized by R package DESeq [48].

### Metabolic pathway score

Twenty-three pathways analyzed in this study include those of choline metabolism [13, 14] as well as energy production and macromolecule biosynthesis, as previously curated for cancer cells [6, 27, 49, 50] outlined in Figure 1 (and detailed in Supplementary Table 1). Assigning an ‘upregulation’ or ‘downregulation’ status to a metabolic pathway requires the contribution of all genes in the pathway. To this end, we selected well-established genes for each pathway, excluding upstream effectors.

In the PDX models, gene expression z-scores were derived from the Mean/SD of all 11 xenograft lines. The pathway score was then calculated from the average z-scores of genes within each pathway/sub-pathway. To compare the VPC cohort with the PDX models, z-scores for VPC gene expression were derived from the Mean/SD of the 14 VPC cancer samples. We obtained enough matched patient tissue for a paired comparison for 2 of the PDX models and original patient tissues (VPCT24:LTL331 and VPCT23:LTL418).

To validate metabolic heterogeneity in a larger cohort of primary untreated prostate cancer specimens (MSKCC), and to ascertain potential clinical predictive value, prostate cancer gene expression was normalized to benign gene expression (Mean/SD) to generate per gene z-scores for the genes expressed in 23 metabolic pathways/sub-pathways (Figure 1).

### Statistical analyses

The pathway scores calculated from the average z-scores of genes within a pathway provide information about the direction of the gene/pathway regulation. In the MSKCC cohort, Wilcoxon rank tests (*p* < 0.05) were applied to determine if pathway scores were significantly up- or down-regulated in tumours compared to benign tissue. Pathways containing fewer than 6 genes were not tested by the Wilcoxon rank test. The MSKCC cohort was also subjected to *t*-test (*p* < 0.05) using log2 gene expression (cancer *vs* benign) and survival curve analysis using pathway z-scores normalized to benign (Kaplan Meier plots) performed in Graphpad 6 Prism (significance determined using the Mantel-Cox test and the Gehan-Breslow test). Cox regression analysis and Spearman correlation were also undertaken to determine if metabolic pathway scores correlated with clinical parameters. Hierarchical clustering was used as previously described [51] using the R language (http://cran.r-project.org/).

## SUPPLEMENTARY MATERIALS TABLES













## Abbreviations

LTL: Living Tumour Lab

## References

[1] Shoag J, Barbieri CE (2016). Clinical variability and molecular heterogeneity in prostate cancer. Asian Journal of Andrology.

[2] Gentric G, Mieulet V, Mechta-Grigoriou F (2016). Heterogeneity in Cancer Metabolism: New Concepts in an Old Field. Antioxidants & Redox Signaling.

[3] Ward Patrick S, Thompson Craig B (2012). Metabolic Reprogramming: A Cancer Hallmark Even Warburg Did Not Anticipate. Cancer Cell.

[4] Hanahan D, Weinberg Robert A (2011). Hallmarks of Cancer: The Next Generation. Cell.

[5] Hay N (2016). Reprogramming glucose metabolism in cancer: can it be exploited for cancer therapy?. Nat Rev Cancer.

[6] Schulze A, Harris AL (2012). How cancer metabolism is tuned for proliferation and vulnerable to disruption. Nature.

[7] Vander Heiden MG, Cantley LC, Thompson CB (2009). Understanding the Warburg Effect: The Metabolic Requirements of Cell Proliferation. Science.

[8] Choi SYC, Collins CC, Gout PW, Wang Y (2013). Cancer-generated lactic acid: a regulatory, immunosuppressive metabolite?. The Journal of Pathology.

[9] Choi SYC, Xue H, Wu R, Fazli L, Lin D, Collins CC, Gleave ME, Gout PW, Wang Y (2016). The MCT4 Gene: A Novel, Potential Target for Therapy of Advanced Prostate Cancer. Clinical Cancer Research.

[10] Boutros PC, Fraser M, Harding NJ, de Borja R, Trudel D, Lalonde E, Meng A, Hennings-Yeomans PH, McPherson A, Sabelnykova VY, Zia A, Fox NS, Livingstone J (2015). Spatial genomic heterogeneity within localized, multifocal prostate cancer. Nat Genet.

[11] Boyd LK, Mao X, Lu YJ (2012). The complexity of prostate cancer: genomic alterations and heterogeneity. Nat Rev Urol.

[12] Ben-Haim S, Ell P (2009). 18F-FDG PET and PET/CT in the Evaluation of Cancer Treatment Response. Journal of Nuclear Medicine.

[13] Bertilsson H, Tessem MB, Flatberg A, Viset T, Gribbestad I, Angelsen A, Halgunset J (2012). Changes in Gene Transcription Underlying the Aberrant Citrate and Choline Metabolism in Human Prostate Cancer Samples. Clinical Cancer Research.

[14] Costello LC, Franklin RB (1991). Concepts of citrate production and secretion by prostate. 1. Metabolic relationships. The Prostate.

[15] Kobus T, Hambrock T, Hulsbergen-van de Kaa CA, Wright AJ, Barentsz JO, Heerschap A, Scheenen TWJ (2011). In Vivo Assessment of Prostate Cancer Aggressiveness Using Magnetic Resonance Spectroscopic Imaging at 3 T with an Endorectal Coil. European Urology.

[16] Schwarzenböck S, Souvatzoglou M, Krause BJ (2012). Choline PET and PET/CT in Primary Diagnosis and Staging of Prostate Cancer. Theranostics.

[17] Lima AR, Bastos MdL, Carvalho M, Guedes de Pinho P (2016). Biomarker Discovery in Human Prostate Cancer: an Update in Metabolomics Studies. Translational Oncology.

[18] Jadvar H (2015). Positron Emission Tomography in Prostate Cancer: Summary of Systematic Reviews and Meta-Analysis. Tomography.

[19] Lin D, Wyatt AW, Xue H, Wang Y, Dong X, Haegert A, Wu R, Brahmbhatt S, Mo F, Jong L, Bell RH, Anderson S, Hurtado-Coll A (2014). High Fidelity Patient-Derived Xenografts for Accelerating Prostate Cancer Discovery and Drug Development. Cancer Research.

[20] Lin D, Xue H, Wang Y, Wu R, Watahiki A, Dong X, Cheng H, Wyatt AW, Collins CC, Gout PW, Wang Y (2014). Next generation patient-derived prostate cancer xenograft models. Asian Journal of Andrology.

[21] Taylor BS, Schultz N, Hieronymus H, Gopalan A, Xiao Y, Carver BS, Arora VK, Kaushik P, Cerami E, Reva B, Antipin Y, Mitsiades N, Landers T (2010). Integrative genomic profiling of human prostate cancer. Cancer cell.

[22] Huan T, Troyer DA, Li L (2016). Metabolite Analysis and Histology on the Exact Same Tissue: Comprehensive Metabolomic Profiling and Metabolic Classification of Prostate Cancer. Scientific Reports.

[23] McDunn JE, Li Z, Adam KP, Neri BP, Wolfert RL, Milburn MV, Lotan Y, Wheeler TM (2013). Metabolomic signatures of aggressive prostate cancer. The Prostate.

[24] Shuster JR, Lance RS, Troyer DA (2011). Molecular preservation by extraction and fixation, mPREF: a method for small molecule biomarker analysis and histology on exactly the same tissue. BMC Clinical Pathology.

[25] Sreekumar A, Poisson LM, Rajendiran TM, Khan AP, Cao Q, Yu J, Laxman B, Mehra R, Lonigro RJ, Li Y, Nyati MK, Ahsan A, Kalyana-Sundaram S (2009). Metabolomic profiles delineate potential role for sarcosine in prostate cancer progression. Nature.

[26] Packer JR, Maitland NJ (2016). The molecular and cellular origin of human prostate cancer. Biochimica et Biophysica Acta (BBA) - Molecular Cell Research.

[27] Hu J, Locasale JW, Bielas JH, O'Sullivan J, Sheahan K, Cantley LC, Heiden MGV, Vitkup D (2013). Heterogeneity of tumor-induced gene expression changes in the human metabolic network. Nat Biotech.

[28] Koppenol WH, Bounds PL, Dang CV (2011). Otto Warburg's contributions to current concepts of cancer metabolism. Nat Rev Cancer.

[29] Tedeschi PM, Markert EK, Gounder M, Lin H, Dvorzhinski D, Dolfi SC, Chan LLY, Qiu J, DiPaola RS, Hirshfield KM, Boros LG, Bertino JR, Oltvai ZN, Vazquez A (2013). Contribution of serine, folate and glycine metabolism to the ATP, NADPH and purine requirements of cancer cells. Cell Death Dis.

[30] Pavlova Natalya N, Thompson Craig B The Emerging Hallmarks of Cancer Metabolism. Cell Metabolism.

[31] Corbin J, Ruiz-Echevarría M (2016). One-Carbon Metabolism in Prostate Cancer: The Role of Androgen Signaling. International Journal of Molecular Sciences.

[32] Fan J, Ye J, Kamphorst JJ, Shlomi T, Thompson CB, Rabinowitz JD (2014). Quantitative flux analysis reveals folate-dependent NADPH production. Nature.

[33] Locasale JW (2013). Serine, glycine and the one-carbon cycle: cancer metabolism in full circle. Nature reviews Cancer.

[34] Martínez-Reyes I, Chandel NS (2014). Mitochondrial one-carbon metabolism maintains redox balance during hypoxia. Cancer discovery.

[35] Joseph R, Bockorny B, Dasanu CA (2014). Methotrexate therapy leading to a rapid progression of a previously indolent prostate cancer: Is immunosuppression to blame?. Journal of Oncology Pharmacy Practice.

[36] Corominas-Faja B, Quirantes-Piné R, Oliveras-Ferraros C, Vazquez-Martin A, Cufí S, Martin-Castillo B, Micol V, Joven J, Segura-Carretero A, Menendez JA (2012). Metabolomic fingerprint reveals that metformin impairs one-carbon metabolism in a manner similar to the antifolate class of chemotherapy drugs. Aging (Albany NY).

[37] Dowling RJO, Goodwin PJ, Stambolic V (2011). Understanding the benefit of metformin use in cancer treatment. BMC Medicine.

[38] Moiseeva O, Xavier Deschênes S, Pollak M, Ferbeyre G (2013). Metformin, aging and cancer. Aging (Albany NY).

[39] Thompson AM (2014). Molecular Pathways: Preclinical Models and Clinical Trials with Metformin in Breast Cancer. American Association for Cancer Research.

[40] Liu W, Hancock CN, Fischer JW, Harman M, Phang JM (2015). Proline biosynthesis augments tumor cell growth and aerobic glycolysis: involvement of pyridine nucleotides. Scientific Reports.

[41] Phang JM, Liu W, Hancock C, Christian KJ (2012). The Proline Regulatory Axis and Cancer. Frontiers in Oncology.

[42] Olivares O, Vasseur S (2016). Metabolic rewiring of pancreatic ductal adenocarcinoma: New routes to follow within the maze. International Journal of Cancer.

[43] Possemato R, Marks KM, Shaul YD, Pacold ME, Kim D, Birsoy K, Sethumadhavan S, Woo HK, Jang HG, Jha AK, Chen WW, Barrett FG, Stransky N (2011). Functional genomics reveal that the serine synthesis pathway is essential in breast cancer. Nature.

[44] Wyatt AW, Mo F, Wang K, McConeghy B, Brahmbhatt S, Jong L, Mitchell DM, Johnston RL, Haegert A, Li E, Liew J, Yeung J, Shrestha R (2014). Heterogeneity in the inter-tumor transcriptome of high risk prostate cancer. Genome Biology.

[45] Dobin A, Davis CA, Schlesinger F, Drenkow J, Zaleski C, Jha S, Batut P, Chaisson M, Gingeras TR (2013). STAR: ultrafast universal RNA-seq aligner. Bioinformatics.

[46] Barnett DW, Garrison EK, Quinlan AR, Strömberg MP, Marth GT (2011). BamTools: a C++ API and toolkit for analyzing and managing BAM files. Bioinformatics.

[47] Anders S, Pyl PT, Huber W (2015). HTSeq—a Python framework to work with high-throughput sequencing data. Bioinformatics.

[48] Anders S, Huber W (2010). Differential expression analysis for sequence count data. Genome Biology.

[49] Harjes U, Bensaad K, Harris AL (2012). Endothelial cell metabolism and implications for cancer therapy. British Journal of Cancer.

[50] Fendt SM, Bell EL, Keibler MA, Davidson SM, Wirth GJ, Fiske B, Mayers JR, Schwab M, Bellinger G, Csibi A, Patnaik A, Blouin MJ, Cantley LC (2013). Metformin Decreases Glucose Oxidation and Increases the Dependency of Prostate Cancer Cells on Reductive Glutamine Metabolism. Cancer Research.

[51] Eisen MB, Spellman PT, Brown PO, Botstein D (1998). Cluster analysis and display of genome-wide expression patterns. Proceedings of the National Academy of Sciences.

